# Supplementary material for: Fusion with Promiscuous Gα16 Subunit Reveals Signaling Bias at Muscarinic Receptors
Source: Int J Mol Sci. 2021 Sep 18;22(18):10089. doi: 10.3390/ijms221810089 (PMC8469978; doi:10.3390/ijms221810089)
Supplement: Supplementary file 1 [file ijms-22-10089-s001.zip › ijms-1285126-supplementary.pdf]

Supplementary material:

Sequences of the fusion proteins

Sequence: $M_1\_G\alpha_{16}$

MNTSAPPAVSPNITVLAPGKGPWQVAFIGITTGLLSLATVTGNLLVLISFKVNTELKTVNNYFLLSLACADLIIGTFSMNLYTTYLLMGHWA
LGTLACDLWLALDYVASNASVMNLLLISFDRYFSVTRPLSYRAKRTPRRAALMIGLAWLVSFVLWAPAILFWQYLVGERTVLAGQCYIQF
LSQPIITFGTAMAAFYLPVTVMCTLYWRIYRETENRARELAALQGSETPGKGGGSSSSSERSQPGAEGSPETPPGRCCRCCRAPRLLQAYSW
KEEEEEDEGSMESLTSSEGEEPGSEVVIKMPMVDPEAQAPTKQPPRSSPNTVKRPTKKGRDRAGKGQKPRGKEQLAKRKTFSLVKEKKAA
RTLSAILLAFILTWTPYNIMVLVSTFCKDCVPETLWELGYWLCYVNSTINPMCYALCNKAFRDTFRLLLLCRWDKRRWRKIPKRPGSVHRT
PTGATRARSLKWRCCPWCLTEDEKAAARVDQEINRILLEQKKQDRGELKLLLLGPGESGKSTFIKQMRIIHGAGYSEEERKGFRPLVYQNIF
VSMRAMIEAMERLQIPFSRPESKHHASLVMSQDPYKVTTFEKRYAAAMQWLWRDAGIRACYERRREFHLLDSAVYYLSHLERITEEGYVP
TAQDVLRSRMPTTGINEYCFSVQKTNLRIVDVGGQKSERKKWIHCFENVIALIYLASLSEYDQCLEENNQENRMKESLALFGTILELPWFKS
TSVILFLNKTDILEEKIPTSHLATYFPSFQGPKQDAEAAKRFILDMYTRMYTGCVDGPEGSKKGARSRRLFSHYTCATDTQNIRKVFKDVRD
SVLARYLDEINLL

Sequence: $M_2\_G\alpha_{16}$

MNNSTNSSNNSLALTSPYKTFEVVFIVLVAGSLSLVTIIGNILVMVSIKVNRHLQTVNNYFLFSLACADLIIGVFSMNLYTLYTVIGYWPLGP
VVCDLWLALDYVVSNASVMNLLIISFDRYFCVTKPLTYPVKRTTKMAGMMIAAAWVLSFILWAPAILFWQFIVGVRTVEDGECYIQFFSNA
AVTFGTAIAAFYLPVIIMTVLYWHISRASKSRIKKDKKEPVANQDPVSPSLVQGRIVKPNNNNMPSSDDGLEHNKIQNGKAPRDPVTENCVQ
GEEKESSNDSTSVSAVASNMRDDEITQDENTVSTSLGHSKDENSKQTCIRIGTKTPKSDSCTPTNTTVEVVGSSGQNGDEKQNIVARKIVKM
TKQPAKKKPPPSREKKVTRTILAILLAFIITWAPYNVMVLINTFCAPCIPNTVWTIGYWLCYINSTINPACYALCNATFKKTFKHLLMCHYKN
TGATRARSLKWRCCPWCLTEDEKAAARVDQEINRILLEQKKQDRGELKLLLLGPGESGKSTFIKQMRIIHGAGYSEEERKGFRPLVYQNIFV
SMRAMIEAMERLQIPFSRPESKHHASLVMSQDPYKVTTFEKRYAAAMQWLWRDAGIRACYERRREFHLLDSAVYYLSHLERITEEGYVPT
AQDVLRSRMPTTGINEYCFSVQKTNLRIVDVGGQKSERKKWIHCFENVIALIYLASLSEYDQCLEENNQENRMKESLALFGTILELPWFKST
SVILFLNKTDILEEKIPTSHLATYFPSFQGPKQDAEAAKRFILDMYTRMYTGCVDGPEGSKKGARSRRLFSHYTCATDTQNIRKVFKDVRDS
VLARYLDEINLL

Sequence: $M_3\_G\alpha_{16}$

MTLHNNSTTSPLFPNISSSWIHSPSDAGLPPGTVTHFGSYNVSRAAGNFSSPDGTTDDPLGGHTVWQVVFIAFLTGILALVTIIGNILVIVSFKV
NKQLKTVNNYFLLSLACADLIIGVISMNLFTTYIIMNRWALGNLACDLWLAIDYVASNASVMNLLVISFDRYFSITRPLTYRAKRTTKRAGV
MIGLAWVISFVLWAPAILFWQYFVGKRTVPPGECFIQFLSEPTITFGTAIAAFYMPVTIMTILYWRIYKETEKRTKELAGLQASGTEAETENF
VHPTGSSRSCSSYELQQQSMKRSNRRKYGRCHFWFTTKSWKPSSEQMDQDHSSSDSWNNNDAAASLENSASSDEEDIGSETRAIYSIVLKL
PGHSTILNSTKLPSSDNLQVPEEELGMVDLERKADKLQAQKSVDDGGSFPKSFSKLPIQLESAVDTAKTSDVNSSVGKSTATLPLSFKEATL
AKRFALKTRSQITKRKRMSLVKEKKAAQTLSAILLAFIITWTPYNIMVLVNTFCDSCIPKTFWNLGYWLCYINSTVNPVCYALCNKTFRTTF
KMLLLCQCDKKKRRKQQYQQRQSVIFHKRAPEQTGATRARSLKWRCCPWCLTEDEKAAARVDQEINRILLEQKKQDRGELKLLLLGPGES
GKSTFIKQMRIIHGAGYSEEERKGFRPLVYQNIFVSMRAMIEAMERLQIPFSRPESKHHASLVMSQDPYKVTTFEKRYAAAMQWLWRDAGI
RACYERRREFHLLDSAVYYLSHLERITEEGYVPTAQDVLRSRMPTTGINEYCFSVQKTNLRIVDVGGQKSERKKWIHCFENVIALIYLASLS
EYDQCLEENNQENRMKESLALFGTILELPWFKSTSVILFLNKTDILEEKIPTSHLATYFPSFQGPKQDAEAAKRFILDMYTRMYTGCVDGPE
GSKKGARSRRLFSHYTCATDTQNIRKVFKDVRDSVLARYLDEINLL

Sequence: $M_4\_G\alpha_{16}$

MANFTPVNGSSGNQSVRLVTSSSHNRYETVEMVFIATVTGSLSLVTVVGNILVMLSIKVNRQLQTVNNYFLFSLACADLIIGAFSMNLYTVY
IIKGYWPLGAVVCDLWLALDYVVSNASVMNLLIISFDRYFCVTKPLTYPARRTTKMAGLMIAAAWVLSFVLWAPAILFWQFVVGKRTVPD
NQCFIQFLSNPAVTFGTAIAAFYLPVVIMTVLYIHISLASRSRVHKHRPEGPKEKKAKTLAFLKSPLMKQSVKKPPPGEAAREELRNGKLEEA
PPPALPPPPRPVADKDTSNESSSGSATQNTKERPATELSTTEATTPAMPAPPLQPRALNPASRWSKIQIVTKQTGNECVTAIEIVPATPAGMRP
AANVARKFASIARNQVRKKRQMAARERKVTRTIFAILLAFILTWTPYNVMVLVNTFCQSCIPDTVWSIGYWLCYVNSTINPACYALCNATF
KKTFRHLLLCQYRNIGTTGATRARSLKWRCCPWCLTEDEKAAARVDQEINRILLEQKKQDRGELKLLLLGPGESGKSTFIKQMRIIHGAGYS
EEERKGFRPLVYQNIFVSMRAMIEAMERLQIPFSRPESKHHASLVMSQDPYKVTTFEKRYAAAMQWLWRDAGIRACYERRREFHLLDSAV
YYLSHLERITEEGYVPTAQDVLRSRMPTTGINEYCFSVQKTNLRIVDVGGQKSERKKWIHCFENVIALIYLASLSEYDQCLEENNQENRMKE
SLALFGTILELPWFKSTSVILFLNKTDILEEKIPTSHLATYFPSFQGPKQDAEAAKRFILDMYTRMYTGCVDGPEGSKKGARSRRLFSHYTCA
TDTQNIRKVFKDVRDSVLARYLDEINLL

Sequence: M_5_$G\alpha_{16}$

MEGDSYHNATTVNGTPVNHQPLERHRLWEVITIAAVTAVVSLITIVGNVLVMISFKVNSQLKTVNNYYLLSLACADLIIGIFSMNLYTTYIL
MGRWALGSLACDLWLALDYVASNASVMNLLVISFDRYFSITRPLTYRAKRTPKRAGIMIGLAWLISFILWAPAILCWQYLVGKRTVPLDEC
QIQFLSEPTITFGTAIAAFYIPVSVMTILYCRIYRETEKRTKDLADLQGSDSVTKAEKRKPAHRALFRSCLRCPRPTLAQRERNQASWSSSRRS
TSTTGKPSQATGPSANWAKAEQLTTCSSYPSSEDEDKPATDPVLQVVYKSQGKESPGEEFSAEETEETFVKAETEKSDYDTPNYLLSPAAAH
RPKSQKCVAYKFRLVVKADGNQETNNGCHKVKIMPCPFPVAKEPSTKGLNPNPSHQMTKRKRVVLVKERKAAQTLSAILLAFIITWTPYNI
MVLVSTFCDKCVPVTLWHLGYWLCYVNSTVNPICYALCNRTFRKTFKMLLLCRWKKKKVEEKLYWQGNSKLTGATRARSLKWRCCPW
CLTEDEKAAARVDQEINRILLEQKKQDRGELKLLLLGPGESGKSTFIKQMRIIHGAGYSEEERKGFRPLVYQNIFVSMRAMIEAMERLQIPFS
RPESKHHASLVMSQDPYKVTTFEKRYAAAMQWLWRDAGIRACYERRREFHLLDSAVYYLSHLERITEEGYVPTAQDVLRSRMPTTGINEY
CFSVQKTNLRIVDVGGQKSERKKWIHCFENVIALIYLASLSEYDQCLEENNQENRMKESLALFGTILELPWFKSTSVILFLNKTDILEEKIPTS
HLATYFPSFQGPKQDAEAAKRFILDMYTRMYTGCVDGPEGSKKGARSRRLFSHYTCATDTQNIRKVFKDVRDSVLARYLDEINLL

Table S1: Parameters of [^{3}H]NMS binding

The equilibrium dissociation constant (K_D) and maximum binding capacity (B_{MAX}) at the cell lines expressing individual subtypes of muscarinic receptors fused with $G\alpha_{16}$ subunit and K_D at wild-type receptors were determined in the saturation experiments. K_D is expressed as a negative logarithm and B_{MAX} is expressed in pmol of binding sites per mg of membrane proteins. Values are mean ± SD from 3 experiments performed in quadruplicates.

	pK_D	B_{MAX} [pmol / mg]	wt mAChRs	pK_D	B_{MAX} [pmol / mg]
**M_1_$G\alpha_{16}$**	9.92 ±0.06	2.50 ±0.14	**M_1**	10.00 ±0.04	5.59 ±0.12
**M_2_$G\alpha_{16}$**	9.27 ±0.06	1.10 ±0.48	**M_2**	9.39 ±0.02	17.34 ±0.47
**M_3_$G\alpha_{16}$**	9.98 ±0.09	0.60 ±0.46	**M_3**	10.03 ±0.01	8.03 ±0.19
**M_4_$G\alpha_{16}$**	9.90 ±0.24	1.13 ±0.64	**M_4**	10.05 ±0.08	9.40 ±0.15
**M_5_$G\alpha_{16}$**	9.91 ±0.25	0.53 ±0.42	**M_5**	9.80 ±0.05	1.4 ±0.03

Table S2: Comparison of low-affinity binding of selected agonists to wt and fused muscarinic receptors

Inhibition constants of indicated muscarinic agonists (K_I) of low-affinity binding to wild-type and $G\alpha_{16}$_fused receptors were calculated according to Eq. 2 from IC_{50} values that were obtained by fitting Eq. 3 to data from competition experiments with [^{3}H]NMS. K_Is are expressed as a negative logarithm. Values are mean ± SD from 3 experiments performed in quadruplicates.

		M_1	M_2	M_3	M_4	M_5
**carbachol**	wt	5.20 ±0.04	5.02 ±0.05	5.15 ±0.04	5.00 ±0.05	5.03 ±0.05
	$G\alpha_{16}$_fused	4.87 ±0.01	4.62 ±0.01	4.77 ±0.02	4.61 ±0.02	4.72 ±0.01
**oxotremorine**	wt	6.43 ±0.04	6.24 ±0.03	6.35 ±0.04	6.21 ±0.03	6.27 ±0.04
	$G\alpha_{16}$_fused	6.61 ±0.01	5.70 ±0.04	6.24 ±0.03	5.86 ±0.02	6.16 ±0.03
**pilocarpine**	wt	5.83 ±0.04	5.63 ±0.04	5.78 ±0.04	5.61 ±0.04	5.68 ±0.04
	$G\alpha_{16}$_fused	5.26 ±0.02	4.52 ±0.01	4.92 ±0.02	4.54 ±0.03	4.88 ±0.04
**JR6**	wt	4.35 ±0.07	4.37 ±0.06	4.43 ±0.03	4.27 ±0.02	4.30 ±0.10
	$G\alpha_{16}$_fused	4.97 ±0.07	5.74 ±0.10	5.07 ±0.04	5.29 ±0.21	5.44 ±0.05
**JR7**	wt	4.95 ±0.07	5.10 ±0.10	5.10 ±0.10	5.10 ±0.10	5.00 ±0.20
	$G\alpha_{16}$_fused	4.34 ±0.05	5.17 ±0.07	4.22 ±0.06	4.82 ±0.03	4.46 ±0.04

Table S3: Parameters of functional response of $G\alpha_{16}$_fused receptors

Parameters of functional response EC_{50} and E'_{MAX} obtained by fitting Eq. 4 to data from measurement of the accumulation of inositol phosphates. EC_{50} is expressed as negative logarithms. Values are means ± SD from 3 independent experiments performed in triplicates.

		**Arecoline**	**Carbachol**	**Furmethide**	**Iperoxo**	**McN-A343**	**NDMC**	**Oxotremorine**	**Pilocarpine**	**Xanomeline**	**JR6**	**JR7**
**M_1_G_{16}**	**pEC50**	6.88 ± 0.01	7.08 ± 0.06	6.52±0.03	8.65 ± 0.03	6.7 ± 0.1	7.61 ± 0.05	8.1 ± 0.05	6.52 ± 0.02	8.5 ± 0.02	n.c	n.c
	**E´MAX**	9.31 ± 0.89	12.75 ± 0.25	10.89±0.09	15.3 ± 0.53	8.02 ± 0.35	9.33 ± 0.16	12.62 ± 0.39	11.01 ± 0.65	12.07 ± 0.11	n.r	n.r
**M2_G_{16}**	**pEC50**	7.14 ± 0.06	6.99 ± 0.06	6.61±0.03	8.65 ± 0.08	6.99 ± 0.04	6.99 ± 0.02	8.22 ± 0.08	6.68 ± 0.02	8.58 ± 0.03	n.c	n.c
	**E´MAX**	18.7 ± 0.46	17.97 ± 0.35	19.13±0.38	27.27 ± 0.64	15.73 ± 0.32	15.13 ± 0.26	22.37 ± 0.64	15.1 ± 0.32	19.13 ± 0.38	n.r	n.r
**M_3_G_{16}**	**pEC50**	7.29 ± 0.01	7.08 ± 0.08	6.17±0.31	9.16 ± 0.1	5.92 ± 0.13	7.61 ± 0.06	8.1 ± 0.05	6.54 ± 0.01	8.5 ± 0.03	n.c	n.c
	**E´MAX**	8.78 ± 0.07	12.97 ± 0.28	10.9±0.15	18.63 ± 5.58	5.87 ± 0.88	9.3 ± 0.06	12.37 ± 0.41	11.27 ± 0.64	11.93 ± 0.34	n.r	n.r
**M_4_G_{16}**	**pEC50**	7.45 ± 0.04	7.38 ± 0.01	6.91±0.01	8.22 ± 0.03	7.16 ± 0.02	7.01 ± 0.02	8.25 ± 0.01	6.66 ± 0.01	8.86 ± 0.01	n.c	n.c
	**E´MAX**	12.14 ± 1.07	12.82 ± 0.57	12.6±1.22	15 ± 0.65	11.33 ± 1.12	10.6 ± 0.86	13.46 ± 0.67	9.94 ± 0.96	12.6 ± 1.22	n.r	n.r
**M_5_G_{16}**	**pEC50**	7.19 ± 0.14	7.03 ± 0.02	6.57±0.03	8.72 ± 0.14	7.09 ± 0.11	7.3 ± 0.03	8.13 ± 0.03	6.6 ± 0.01	8.54 ± 0.01	n.c	n.c
	**E´MAX**	8.44 ± 0.09	15.3 ± 0.2	14.97±0.15	11.3 ± 0.43	6.59 ± 0.16	12.2 ± 0.15	17.43 ± 0.18	13 ± 0.38	15.57 ± 0.2	n.r	n.r

n.c., not calculated; n.r., no response;

Suplementary data analysis

Quantifiaction of agonist bias

Operational efficacy (τ) and agonist equilibrium dissociation constant (K_A) of tested agonist and reference agonist are sufficient to quantify the agonism of a given ligand. The parameter K_A is specific to a combination of ligand and receptor. The parameter τ is specific to a combination of ligand and signalling system. A parameter for characterizing agonism for a given system can be defined as a "transduction coefficient" and is equal to the logarithm of τ to K_A ratio, $\log(\tau/K_A)$. The relative efficiency of two agonists producing activation of a given pathway is quantified as the difference between value of transduction coefficient of tested ligand and transduction coeficient of reference ligand, $\Delta\log(\tau/K_A)$. For a biased ligand, $\Delta\log(\tau/K_A)$ of the biased pathway is greater than $\Delta\log(\tau/K_A)$ of another (reference) pathway. Analogically, two systems of a single signaling pathway and two different receptors can be compared. A ligand that has greater $\Delta\log(\tau/K_A)$ at one system (receptor) than at other is biased to a given system (receptor). Then, the ligand bias for receptor A over receptor B is quantified according to Eq. 1.

$$Bias\ factor\ =\ 10^{\Delta\log\left(\frac{\tau}{K_A}\right)_{receptor\ A}\ -\ \Delta\log\left(\frac{\tau}{K_A}\right)_{receptor\ B}}$$ Eq. S1

Table S4: Quantifiaction of agonist bias via ΔΔlog(τ/KA).

Values of τ and K_A were calculated according to Eq. 5, 6 and are summarized in Table 2 of the main manuscript. First, the transduction coefficient, log(τ/K_A), was calculated. Then, the relative efficiency to the reference agonist carbachol as difference in transduction coefficients, Δlog(τ/K_A). Ligand bias at two different G16_fused receptors equal to differences of relative efficiencies on these pathways, ΔΔlog(τ/K_A), was used for determination of agonist bias according Eq.S1. Parameters Δlog(τ/K_A); ΔΔlog(τ/K_A) and bias factor$10^{\Delta\Delta\log(\tau/KA)}$ are summarized in table S4 a,b,c.

a)

Δ**log**(τ/K_A)	**Arecoline**	**Furmethide**	**Iperoxo**	**McN-A343**	**NDMC**	**Oxotremorine**	**Pilocarpine**	**Xanomeline**
**M1**	-0.294 ± 0.034	-0.632 ±0.018	1.690 ±0.0425	-0.544 ± 0.06	**0.381** ± 0.0295	1.011 ± 0.0385	-0.633 ± 0.03	1.402 ± 0.0135
**M2**	**0.176** ± 0.049	**-0.347** ±0.032	1.854 ±0.1535	-0.055 ± 0.0305	-0.071 ± 0.018	**1.338** ± 0.0805	-0.383 ± 0.02	**1.623** ± 0.032
**M3**	0.070 ± 0.007	-0.996 ±0.160	**2.263** ±0.488	-1.466 ± 0.088	**0.364** ± 0.0315	0.998 ± 0.0385	-0.610 ± 0.0245	1.378 ± 0.026
**M4**	0.042 ± 0.056	-0.476 ±0.047	0.922 ±0.0425	-0.275 ± 0.045	-0.458 ± 0.035	0.895 ± 0.0295	-0.837 ± 0.032	1.475 ± 0.047
**M5**	-0.024 ± 0.073	-0.471 ±0.02	1.625 ±0.0855	-0.238 ± 0.0595	0.169 ± 0.0195	1.160 ± 0.023	-0.508 ± 0.0165	1.517 ± 0.0125

b)

ΔΔ**log**(τ/K_A)	**Arecoline**	**Furmethide**	**Iperoxo**	**McN-A343**	**NDMC**	**Oxotremorine**	**Pilocarpine**	**Xanomeline**
**M2 vs M1**	0.470 ± 0.049	0.285 ± 0.032	0.164 ± 0.1535	0.489 ± 0.06	-0.452 ± 0.0295	0.327 ± 0.0805	0.250 ± 0.03	0.221 ± 0.032
**M3 vs M1**	0.364 ± 0.034	-0.364 ± 0.160	0.574 ± 0.488	-0.922 ± 0.088	-0.018 ± 0.032	-0.013 ± 0.039	0.024 ± 0.030	-0.025 ± 0.026
**M4 vs M1**	0.337 ± 0.056	0.156 ± 0.047	-0.768 ± 0.043	0.269 ± 0.060	-0.839 ± 0.035	-0.117 ± 0.039	-0.204 ± 0.032	0.072 ± 0.047
**M5 vs M1**	0.270 ± 0.073	0.160 ± 0.020	-0.065 ± 0.086	0.306 ± 0.060	-0.212 ± 0.030	0.149 ± 0.039	0.125 ± 0.030	0.115 ± 0.014
**M3 vs M2**	-0.105 ± 0.049	-0.649 ± 0.1595	0.410 ± 0.488	-1.411 ± 0.088	0.435 ± 0.0315	-0.339 ± 0.0805	-0.226 ± 0.0245	-0.246 ± 0.032
**M4 vs M2**	-0.133 ± 0.056	-0.129 ± 0.047	-0.931 ± 0.154	-0.220 ± 0.045	-0.387 ± 0.035	-0.443 ± 0.081	-0.454 ± 0.032	-0.149 ± 0.047
**M5 vs M2**	-0.200 ± 0.073	-0.125 ± 0.032	-0.229 ± 0.154	-0.183 ± 0.060	0.240 ± 0.020	-0.178 ± 0.081	-0.125 ± 0.020	-0.106 ± 0.032
**M4 vs M3**	-0.028 ± 0.056	0.520 ± 0.160	-1.341 ± 0.488	1.190 ± 0.088	-0.822 ± 0.035	-0.104 ± 0.039	-0.227 ± 0.032	0.097 ± 0.047
**M5 vs M3**	-0.094 ± 0.073	0.525 ± 0.160	-0.639 ± 0.488	1.228 ± 0.088	-0.194 ± 0.032	0.162 ± 0.039	0.101 ± 0.025	0.140 ± 0.026
**M5 vs M4**	-0.066 ± 0.073	0.004 ± 0.047	0.703 ± 0.086	0.038 ± 0.060	0.627 ± 0.035	0.266 ± 0.030	0.328 ± 0.032	0.043 ± 0.047

c)

$10^{\Delta\Delta \log(\tau/KA)}$	Arecoline	Furmethide	Iperoxo	McN-A343	NDMC	Oxotremorine	Pilocarpine	Xanomeline
**M2 vs M1**	2.95 ±0.31	1.93 ±0.22	1.46 ±1.37	3.08 ± 0.38	0.35 ± 0.02	2.12 ± 0.52	1.78 ± 0.21	1.66 ± 0.24
**M3 vs M1**	2.31 ±0.21	0.43 ±0.19	3.75 ±3.19	0.12 ± 0.01	0.96 ± 1.73	0.97 ± 2.93	1.06 ± 1.35	0.94 ± 0.99
**M4 vs M1**	2.17 ±0.36	1.43 ±0.43	0.17 ±0.01	1.86 ± 0.41	0.14 ± 0.01	0.76 ± 0.25	0.63 ± 0.10	1.18 ± 0.77
**M5 vs M1**	1.86 ±0.50	1.45 ±0.18	0.86 ±1.13	2.02 ± 0.40	0.61 ± 0.09	1.41 ± 0.36	1.33 ± 0.32	1.30 ± 0.15
**M3 vs M2**	0.785 ±0.366	0.22 ±0.06	2.57 ±3.06	0.04 ± 0.00	2.72 ± 0.20	0.46 ± 0.11	0.59 ± 0.06	0.57 ± 0.07
**M4 vs M2**	0.736 ±0.307	0.74 ±0.27	0.12 ±0.02	0.60 ± 0.12	0.41 ± 0.04	0.36 ± 0.07	0.35 ± 0.02	0.71 ± 0.22
**M5 vs M2**	0.632 ±0.229	0.75 ±0.19	0.59 ±0.40	0.66 ± 0.21	1.74 ± 0.14	0.66 ± 0.30	0.75 ± 0.12	0.78 ± 0.24
**M4 vs M3**	0.938 ±1.866	3.31 ±1.02	0.05 ±0.02	15.51 ± 1.15	0.15 ± 0.01	0.79 ± 0.29	0.59 ± 0.08	1.25 ± 0.61
**M5 vs M3**	0.805 ±0.619	3.35 ±1.02	0.23 ±0.18	16.91 ± 1.21	0.64 ± 0.10	1.45 ± 0.35	1.26 ± 0.31	1.38 ± 0.26
**M5 vs M4**	0.858 ±0.937	1.01 ±10.71	5.05 ±0.61	1.09 ± 1.73	4.24 ± 0.24	1.84 ± 0.20	2.13 ± 0.21	1.10 ± 1.22

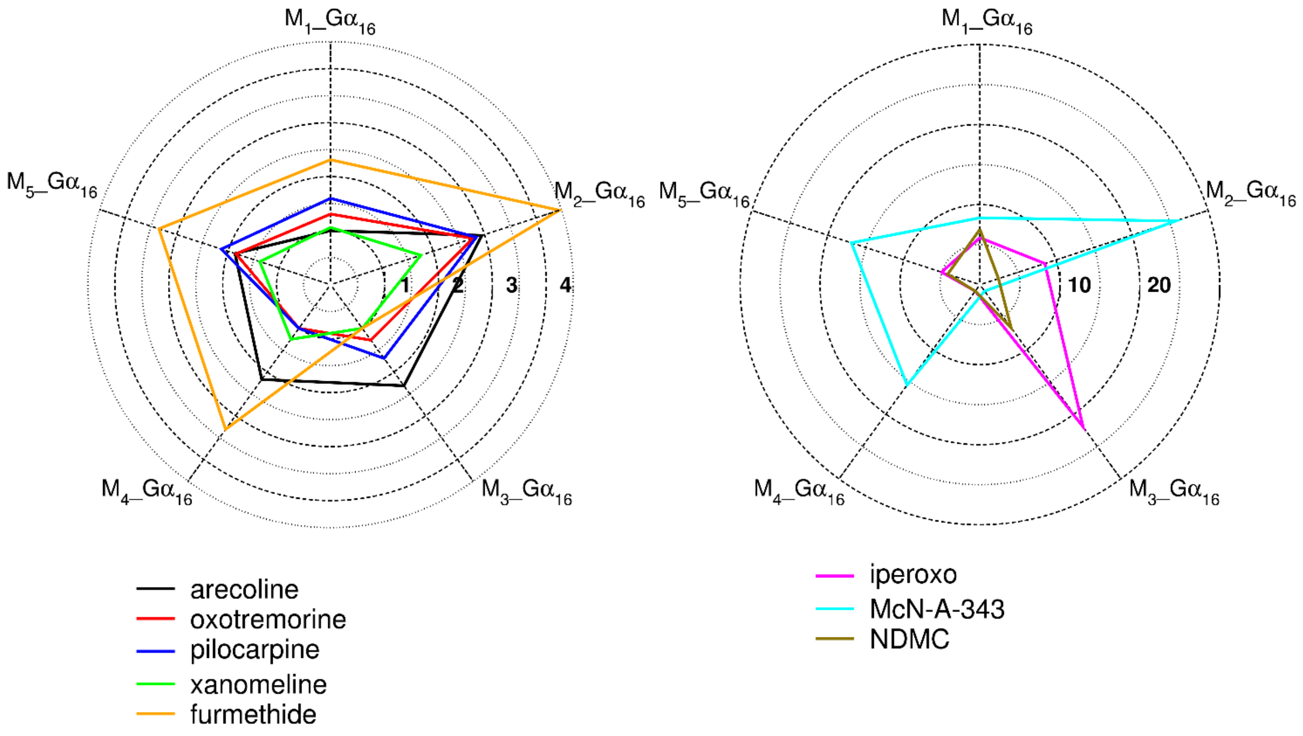

Figure S1: Polar plot of agonist bias factors.

Bias factors of individual agonists in the given receptor system $10^{\Delta\Delta\log(\tau/KA)}$ calculated according to Eq. S1 are plotted. Values are expressed as ratios of bias factors to bias factor at receptor with the lowest activity for given agonist (Arecoline M1; Furmethide, McN-A-343, Xanomeline M3; NDMC, Oxotremorine, Iperoxo, Pilocarpine M4).

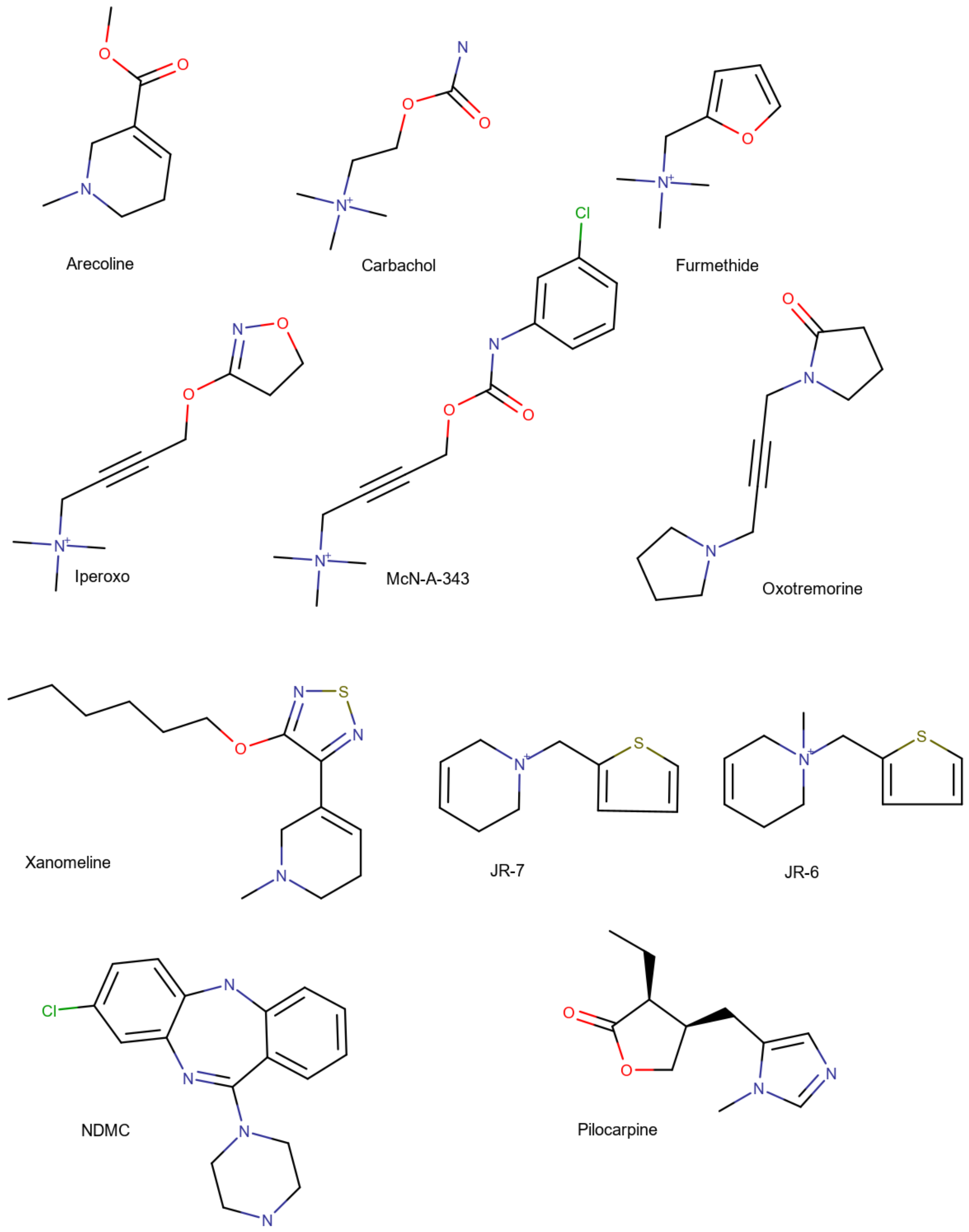

Figure S2: Structures of used agonists

Arecoline: methyl 1-methyl-3,6-dihydro-2H-pyridine-5-carboxylate; **Carbachol**: 2-carbamoyloxyethyl(trimethyl)azanium; **Furmethide**: furan-2-ylmethyl(trimethyl)azanium; **Iperoxo**: 4-(4,5-dihydro-1,2-oxazol-3-yloxy)but-2-ynyl-trimethylazanium; **McN-A-343**: 4-(trimethylazaniumyl)but-2-yn-1-yl N-(3-chlorophenyl)carbamate; **Oxotremorine**: 1-[4-(pyrrolidin-1-yl)but-2-yn-1-yl]pyrrolidin-2-one; **Xanomeline**: 3-hexoxy-4-(1-methyl-3,6-dihydro-2H-pyridin-5-yl)-1,2,5-thiadiazole; **JR6**: 1-[(thiophen-2-yl) methyl]-1,2,3,6-tetrahydropyridin-1-ium; **JR7**: 1-methyl-1-[(thiophen-2-yl)methyl]-1,2,3,6-tetra hydropyridin-1-ium; **N-desmethylclozapine (NDMC)**: 3-chloro-6-piperazin-1-yl-11H-benzo[b][1,4]benzodiazepine; **Pilocarpine**: (3S,4R)-3-ethyl-4-[(1-methyl-1H-imidazol-5-yl)methyl]oxolan-2-one.

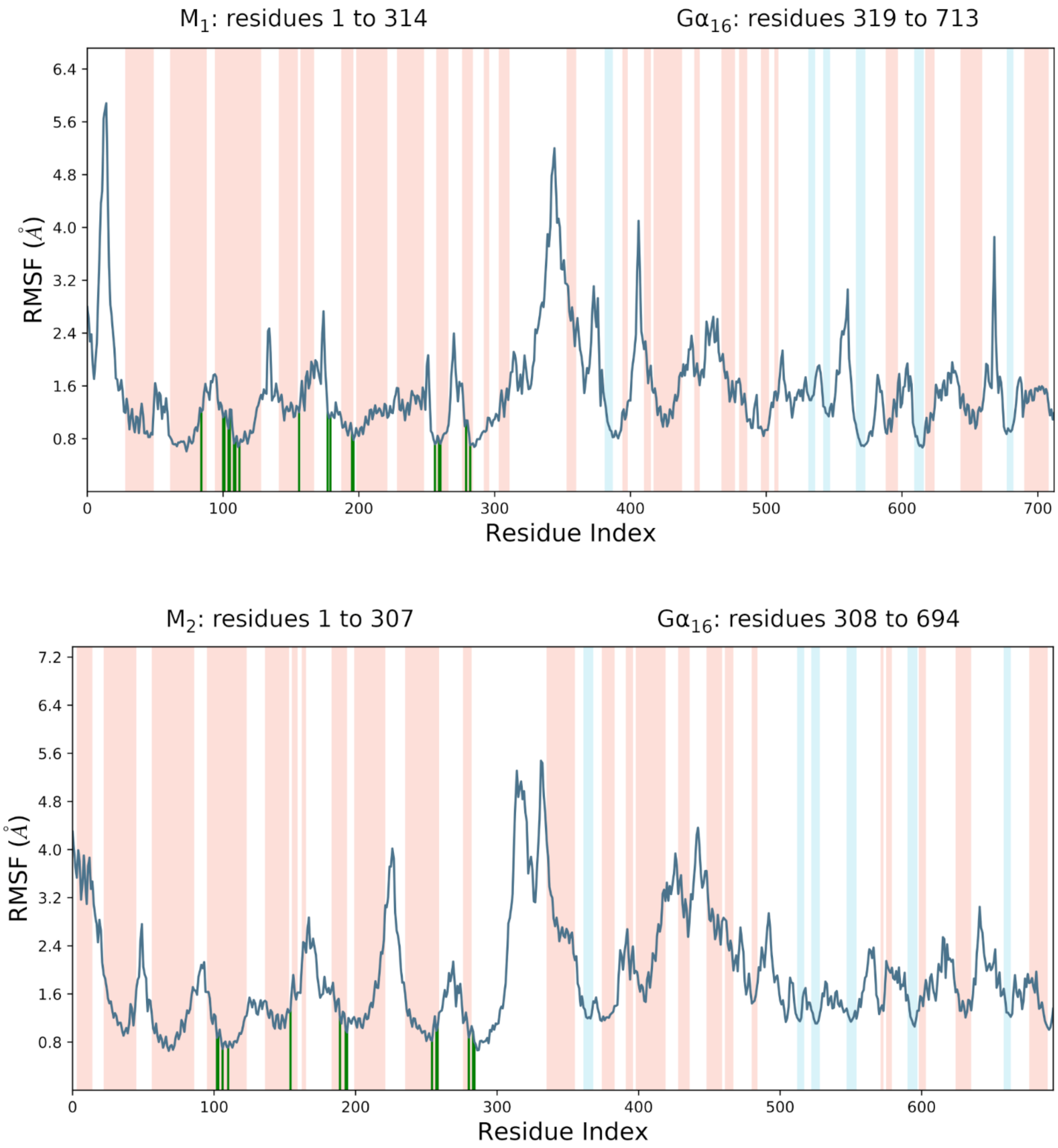

Figure S3: **The Root Mean Square Fluctuation (RMSF) of fusion proteins.**

RMSF in A of individual residues of M_1_Gα_{16} (top) and M_2_Gα_{16} (bottom) from 120-ns simulation of molecular dynamics. Secondary Structure Elements: Alpha-helical and beta-strand regions are highlighted in red and blue backgrounds, respectively. These regions are defined by helices or strands that persist over 70% of the entire simulation. Ligand Contacts: Protein residues that interact with the ligand are marked with green-colored vertical bars.
